# Diversity and conservation of amphibians and reptiles of a protected and heavily disturbed forest of central Mexico

**DOI:** 10.3897/zookeys.830.31490

**Published:** 2019-03-14

**Authors:** Aníbal H. Díaz de la V ga-Pérez, Víctor H. iménez-Arcos, Eric Centenero-Alcalá, Fausto R. Méndez-de la ruz, Andre Ngo

**Affiliations:** 1 Consejo Nacional de Ciencia y Tecnología-Centro Tlaxcala de Biología de la Conducta, Universidad Autónoma de Tlaxcala. Carretera Tlaxcala-Puebla km 1.5, C.P. 90062, Tlaxcala, Mexico; 2 Laboratorio de Ecología, UBIPRO, FES Iztacala Universidad Nacional Autónoma de México. Av. de los Barrios No. 1, Los Reyes Iztacala, C.P. 54090, Tlalnepantla, Mexico; 3 Naturam Sequi A.C. 16 de septiembre 43, Ciudad de los niños, C.P. 53450, Naucalpan, Mexico; 4 Laboratorio de Herpetología, Departamento de Zoología, Instituto de Biología, Universidad Nacional Autónoma de México, Ciudad Universitaria, C.P. 04510, Coyoacán, Ciudad de México, Mexico; 5 Little Ray’s Reptile Zoo, 869 Barton St E, Hamilton, ON L8L 3B4, Canada

**Keywords:** Herpetofauna, natural protected area, high mountain ecosystem, β-diversity

## Abstract

The high loss rate of forest ecosystem by deforestation in the Trans-Mexican Volcanic Belt is one of the principal ecological problems of central Mexico, even in natural protected areas. We compiled a checklist and determined β-diversity indexes of amphibians and reptiles of the highly disturbed protected area, La Malinche National Park (LMNP) in Mexico, to determine the principal habitats for herpetofaunal conservation. After our extensive eight-year field sampling, we documented 28 species (nine amphibians and 19 reptiles), representing 11 families and 18 genera; four of these species are new records for LMNP. Of the species, 89% are endemic to Mexico. The IUCN Red List considers 22 species as Least Concern, one as Near Threatened, and four as Vulnerable. Meanwhile, the Environmental Viability Scores categorize three species as low vulnerability, 15 as medium, and 10 as high. According to the Mexican list of protected species, eight species are under Special Protection and nine are considered Vulnerable. The dissimilarity index between habitat types (βsør) in both groups is high, principally due to the environmental gradient generated by the altitudinal range. *Abies* and Pine forest are high diversity areas for amphibians and reptiles, respectively, and must be considered for special protection. LMNP hosts more than 60% of the herpetofauna of Tlaxcala and is the principal “conservation island” for this state. Therefore, based on the percentage of state species represented, endemism and the current social and ecological problems, additional efforts that involve the local communities to protect the biodiversity of this National Park are necessary.

## Introduction

**Figure 1.**
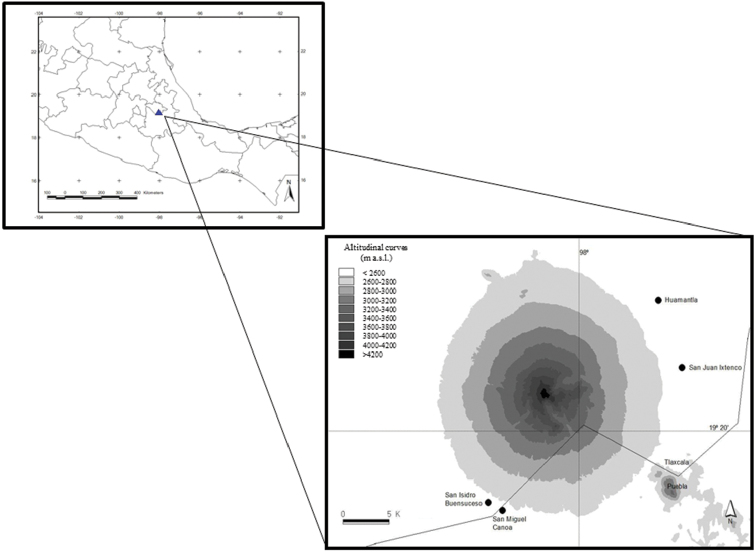
Study area. Geographic delimitation and altitudinal curves of the volcano La Malinche, between the Mexican states of Tlaxcala and Puebla.

Mexico presents the highest richness of amphibians and reptile species in Mesoamerica (Mexico, Belize, Guatemala, El Salvador, Honduras, Nicaragua, Costa Rica, and Panama) not solely due to the sheer size of the country (Wilson and Johnson 2010). The orography of Mexico is one of the factors that contributes to the high biodiversity of these groups of vertebrates. According to Flores-Villela et al. (2010), 131 reptiles and 217 amphibian species inhabit the central mountains of Mexico, including a southern region of the central plain, the Sierra Madre del Sur, and the Trans-Mexican Volcanic Belt (TMVB). The TMVB crosses from the west to the east coast through the center of Mexico, and is formed by many active and inactive volcanoes (Ferrari 2000). This region hosts a high diversity of species, is one of the most important transition zones between two biogeographic regions (Neotropical and Nearctic) and is where the biotas overlap (Morrone 2010). The TMVB holds about 50% of the microendemic amphibian species reported for the whole country (Ochoa-Ochoa et al. 2011). Worryingly, approximately 1% of the original forests of the TMVB disappear every year, and 70% of the natural ecosystems have been transformed into agrosystems and settlements (Toledo et al. 1989, Challenger 1998, Arriaga et al. 2000, Sánchez-Cordero et al. 2005). Within the TMVB lies La Malinche (also called Matlalcuéyatl) which, at 4461 m elevation is the 6^th^ highest peak and the most isolated volcano in Mexico (Fig. 1).

This volcano and the surrounding area were designated La Malinche National Park (LMNP) in 1938. Despite this designation, this protected natural area is still subject to numerous ecological and social problems; nearly 60% of its original vegetation has been removed by local communities for crops and to expand their urban settlements (Villers-Ruiz and López-Blanco 2004). The habitat from 2400 to 2800 m elevation is deteriorated by human activity, such as agriculture, open cattle grazing, farming, fire, and induced grassland (Villers-Ruiz et al. 2006). These human activities are considered the greatest threat to the conservation of biodiversity in high mountain ecosystems, and LMNP is no exception. Because of these activities, this national park presents a high rate of deforestation (20 ha per year) and 77% of the vegetation has deteriorated since it was designated a national park (Díaz-Ojeda 1992, Vargas-Márquez 1997, SEMARNAT 2013). In addition, misinformation and the local beliefs of the people inhabiting the lowlands of LMNP promote the death of many amphibians and reptiles every day.

Previous studies in LMNP have documented 23 reptile and amphibian species (15 and eight species, respectively) in the area. In 1978, Sánchez-de Tagle performed the first herpetofaunal assessment of LMNP, reporting two amphibian species and seven reptile species. Two years later, in a study of Tlaxcala´s herpetofauna, Sánchez-Herrera (1980) determined that 12 species occur in LMNP (two amphibians and 10 reptiles) and four additional species in the surrounding areas. Subsequently, Sánchez-Herrera and López-Ortega (1987) added one lizard species (*Aspidosceliscostata*) to the documented herpetofauna of LMNP. This species had been previously misidentified and reported as *Cnemidophorusgularis* by Sánchez-Herrera (1980). Afterwards, Sanchez-Aguilar (2005), based on a year of fieldwork combined with an analysis of the literature, published a further list of herpetofauna for LMNP identifying 21 species: seven amphibians (two without specific identification) and 14 reptiles; five of these species were new records (*Pseudoeuryceabellii* [*Isthmurabellii*], *Eumeceslynxe* [*Plestiodonlynxe*], *Sceloporusmegalepidurus*, *S.scalaris* and *Storeriastorerioides*). A year later, Gómez-Álvarez and Reyes-Gómez (2006) documented 15 species of herpetofauna (four amphibians and 11 reptiles) from nine years in a single transect, from 2600 to 3500 m elevation on the north-facing slope of LMNP in Tlaxcala. They found three species not previously reported from the area (*Hylaeximia* [*Dryophyteseximius*], *Ambystomaaltamiranoi* and *Thamnophiseques*), eight species in common with the first herpetofaunal list of Sánchez-de Tagle (1978) and 12 with that of Sanchez-Aguilar (2005). Nevertheless, *Ambystomaaltamiranoi* was a misidentification by Gómez-Álvarez and Reyes-Gómez (2006) and was correctly identified as *A.velasci* by Ramírez-Bautista et&nbsp;al. (2009). Later, Fernández et al. (2006) identified two presumed new species records for LMNP (*Chiropterotriton* sp. and *Storeriastorerioides*); however, these two species had been previously published by Sánchez-Aguilar (2005) (Table 1).

**Table 1.** Checklist of amphibians and reptiles of La Malinche National Park, Mexico. We provide the state presence, habitat type (Cropland = C, Pine-Oak forest = POF, Pine forest = PF, *Abies* forest = AF, Alpine grassland = AG, Oak forest = OF, Human constructions = HC, Pine-*Alnus* forest = PAF), IUCN status (Least Concern = LC, Near Threatened = NT, Vulnerable = V, Endangered = E, Critically Endangered = CE) according to the IUCN Red List, the Environmental Vulnerability Score (The EVS range is broken into the following three categories: low (3–9), medium (10–13), and high vulnerability (14–19) from Wilson et al. (2013a, b), and the conservation status in Mexico (subject to special protection = Pr, Threatened =A, Danger of extinction = P, and Not listed = NL) according to SEMARNAT (NOM 059-2010). Source refers to the origin of the information: 1) Sánchez-de Tagle (1978); 2) Sánchez-Herrera (1980); 3) Sánchez-Herrera and López-Ortega (1987); 4) Sánchez-Aguilar (2005); 5) Fernández et al. (2006); 6) Gómez-Álvarez and Reyes-Gómez (2006); 7) This study.

	State	Habitat type	IUCN status	EVS score	NOM 059 2010	Source
Class Amphibia						
Order Caudata						
Family Ambistomatidae						
Ambystomavelasci*	P/T	C, HC	LC	10	Pr	6,7
Family Plethodontidae						
Aquiloeuryceacephalica*	P/T	AF	NT	14	A	4, 7
Chiropterotritonorculus*	P/T	AF	VU	18	NL	4,5,7
Isthmurabellii*	T	–	VU	12	A	4
Pseudoeuryceagadovii*	P/T	AF	VU	13	Pr	1,2,4,7
Pseudoeurycealeprosa*	P/T	POF, PF, AF, PAF	LC	16	A	1,2,4,6,7
Order Anura						
Family Hylidae						
*Dryophyteseximius* *	P/T	C, POF, PF, PAF	LC	10	NL	6,7
Dryophytesplicatus*	P/T	C, PF, HC	LC	11	A	7
Family Scaphiopodidae						
* Spea multiplicata *	P/T	C, OF, PF, HC, PAF	LC	6	NL	4,6,7
Class Reptilia						
Order Squamata						
Suborder Lacertilia						
Family Anguidae						
Barisiaimbricata*	P/T	C, POF, PF, AF, AG, OF, PAF	LC	14	Pr	1,4,6,7
Family Phrynosomatidae						
Phrynosomaorbiculare*	P/T	C, POF, PF, AG	LC	12	A	1,4,6,7
Sceloporusaeneus*	P/T	C, POF, PF, AG, HC, PAF	LC	13	NL	2,4,6,7
Sceloporusbicanthalis*	P/T	PF, AG, PAF	LC	13	NL	1,4,6,7
* Sceloporus grammicus *	P/T	C, POF, PF, AF, AG, OF, HC, PAF	LC	9	Pr	1,2,4,6,7
Sceloporusmegalepidurus*	T	C	VU	14	Pr	2,4
Sceloporusscalaris*	T	AG	LC	12	NL	4,6
Sceloporusspinosus*	T	C, HC	LC	12	NL	7
Family Scincidae						
Plestiodonbrevirostris*	P/T	C, POF, PF, AF, HC	LC	11	NL	2,4,6,7
Plestiodonlynxe*	T	–	LC	10	Pr	4
Family Teiidae						
Aspidosceliscostata*	T	C, OF, HC	LC	11	Pr	2,3,7
Order Squamata						
Suborder Serpentes						
Family Colubridae						
Conopsislineata*	P/T	C, POF, PF	LC	13	NL	7
Pituophisdeppei*	P/T	C	LC	14	A	7
Salvadorabairdi*	T	C	LC	15	Pr	7
Family Natricidae						
Storeriastorerioides*	P/T	C, POF, OF, PF	LC	11	NL	4,5,7
* Thamnophis eques *	T	C, PF	LC	8	A	6
Thamnophisscalaris*	P/T	C, POF, PF, AF, AG, OF, HC, PAF	LC	14	A	1,2,4,6,7
Family Viperidae						
Crotalusravus*	P/T	C, POF, PF, AG, HC, PAF	LC	14	A	1,2,4,6,7
Crotalustriseriatus*	P/T	POF, PF, AF, AG, OF, PAF	LC	16	NL	1,2,4,6,7

* endemic of Mexico, – no information.

Amphibians and reptiles are ideal bio-indicators of the ecosystem health due to their high sensitivity to environmental change; nevertheless, they are not the most common study groups (Welsh and Droege 2001, Siddig et al. 2016). Additionally, the absence of recent biodiversity studies in natural protected areas, like LMNP, and the high rate of habitat change, necessitate the urgent compilation of information that allows assessment of the status of the herpetofauna of these conservation areas. Therefore, our objective is to provide an analysis of amphibian and reptile species richness and a biodiversity analysis of LMNP, to identify high-diversity areas on which to focus conservation efforts. Moreover, we perform a dissimilarity analysis among habitat types, as an indicator of β-diversity, in order to evaluate the herpetofaunal community. This effort promotes their study and provides a guide to future conservation strategies, by providing accurate information to government agencies.

## Materials and methods

### Study site

LMNP is found between the Mexican states of Tlaxcala (70%) and Puebla (19.240195N; -98.034472W). It covers an area of 46,112 ha, ranges in elevation from 2400 to 4461 m, and is largest national park in the TMVB (SEMARNAT 2013). LMNP is a high mountain ecosystem, and the climate and the vegetation community changes according to altitude, air temperature, and humidity. Villers-Ruiz et al., 2006 analyzed the vegetation of LMNP according to elevation gradient and proposed that from 2400 to 2800 m elevation, is the most deteriorated habitat, affected by activities such as agriculture, cattle grazing, fire, and induced grassland, presenting a semiarid climate with a temperature between 14 and 16 °C (SEMARNAT 2013). Above that, from 2800 to 3000 m, there are patches of Oak and Pine forest, agriculture, cattle grazed land, and induced grassland, presenting a sub-humid climate with a temperature between 12 and 18 °C. Between 3000 and 4000 m is a semi-cold climate, with temperatures ranging from 5 to 12 °C, where abundant communities of Pine, *Alnus*, and *Abies* forest exist. Above 4000 m, only a few patches of *Juniperusmonticola* are present as shrubs and alpine grassland dominates in a cold climate with temperatures ranging from 2 to 5 °C.

### Data collection

We generated this list of the amphibians and reptiles of the LMNP from: 1) available herpetofaunal literature: Sánchez-de Tagle (1978), Sánchez-Herrera (1980), Sánchez-Herrera and López-Ortega (1987), Sanchez-Aguilar (2005), Gómez-Álvarez and Reyes-Gómez (2006), Fernández et al. (2006); 2) databases from national scientific collections to which we had access: Colección Herpetológica, Museo de Zoología “Alfonso L. Herrera”, Facultad de Ciencias UNAM (MZFC-UNAM); Colección Nacional de Anfibios y Reptiles, Instituto de Biología UNAM (CNAR); 3) Global Biodiversity Information Facility (GBIF, http://doi.org/10.15468/dl.n4pvrm); and, most importantly; 4) through eight years of fieldwork in LMNP (2010–2018).

We performed an average of seven-field visits per year for five days (four to five people per visit). We included dry and wet seasons all around the volcano slopes and in eight different habitat types (community vegetation and human modification types, see Results section). We made at least one visit to each community vegetation and human modification type each season every year. The sampling was homogeneous among slopes and vegetation types. We used direct capture methods with diurnal and nocturnal searching (nocturnal surveys were less frequent because LMNP is highly insecure). All species previously reported in the literature from field sampling efforts were included in the present list, even if we could not confirm the record by direct observation or by vouchers in a scientific collection. We deposited images of vouchers of new species records in the Instituto de Biología, UNAM (CNAR-IB) scientific collection.

### Threatened status of species and β-diversity analysis

We included the conservation status of each species according to: 1) the IUCN Red List 2018; 2) environmental viability scores (EVS) from Wilson et al. (2013a, b); and 3) the Mexican species´ protection list (SEMARNAT, NOM 059-2010). Vegetation type (presence/absence) was identified for all species following Villers-Ruiz et al. (2006). We also include human constructions as a habitat type.

We use the Sørensen dissimilarity index (β_sør_) as our approach to determine beta-diversity (Sørensen 1948). The β_sør_ quantifies the proportion of species shared between two communities incorporating both true spatial turnover (i.e. taxonomic turnover) and differences in richness by nesting (Koleff et al. 2003, Baselga 2010). We performed the β_sør_ analysis for amphibians, reptiles, and both groups together (herpetofauna). To estimate β_sør_ we performed a dissimilitude linkage matrix using the software R ver. 3.5.0 (R Development Core Team, 2008) with the ‘betapart’ package (Baselga and Orme 2012). Because β_sør_ is composed of the sum of the component of the net taxonomic turnover (β_sim_) and the difference between communities by nesting in the species composition (β_nes_), we present both components in the Suppl. material 1: Tables S1–S6). In addition, we analyzed the proportion of the LMNP´s herpetofauna against that found in the states of Tlaxcala and Puebla, and Mexico as a whole.

## Results

### Species richness

The herpetofauna of LMNP includes 28 species: nine amphibians (six caudates and three anurans) and 19 reptiles (11 lizards and eight snakes). These taxa represent 11 families (four amphibians and seven reptiles) and 18 genera (seven amphibians and 11 reptiles). All the species of the present list were found in Tlaxcala, and eight of these species were only recorded from this state (one amphibian and seven reptiles) (Table 1).

We added four previously undocumented species from LMNP. Three of these new records, the frog, *Dryophytesplicatus* (CNAR-IB-RF 515-516), the lizard, *Sceloporusspinosus* (CNAR-IB-RF 517-518), and the snake *Salvadorabairdi* (observation), were made by direct capture or observations in the field (Table 1). The fourth new record, the snake *Pituophisdeppei*, was not directly observed. However, resident people have seen this species sporadically; moreover, there are precise records of this species in the agricultural fields close to the lowest region of LMNP (~6 km straight line distance, Santa Ana Chiautempan Municipality). Because of this, we included *P.deppei* in the LMNP herpetofaunal list. Additionally, we corroborated the presence of *Conopsislineata* (CNAR-IB-RF 519-520) that has been recorded with imprecise locality near to LMNP (ENCB, 0.5 km S, 6.5 km E San Francisco Tetlanhocan). There were five species, previously reported from LMNP, that we could not verify through fieldwork, photographs, or in scientific collections. These were the amphibian, *Isthmurabellii*, and the reptiles, *Sceloporusmegalepidurus, S.scalaris, Plestiodonlynxe*, and *Thamnophiseques.* Because they were previously documented from LMNP, they are included in our final LMNP herpetofaunal list and included in the analyses where possible.

### Threatened status of species

Four of the reptile and amphibian species found in LMNP are considered Vulnerable according to the IUCN Red List (three amphibians and one reptile); one Near Threatened (the salamander *Aquiloeuryceacephalica*); and 23 Least Concern (five amphibians and 18 reptiles) (Table 1). Using Wilson et al.’s (2013a, b) EVS score, three species are considered to have low vulnerability (one amphibian and two reptiles); 15 with medium vulnerability (five amphibians and 10 reptiles); and 10 are highly vulnerable to extinction (three amphibians and seven reptiles) (Table 1). However, according to the Mexican Species Protection List (SEMARNAT 2010), nine species are Threatened (four amphibians and five reptiles), eight are Subject to Special Protection (two amphibians and six reptiles), and 11 are not listed under any protection category (three amphibians and eight reptiles) (Table 1).

### β-diversity analysis

**Figure 2.**
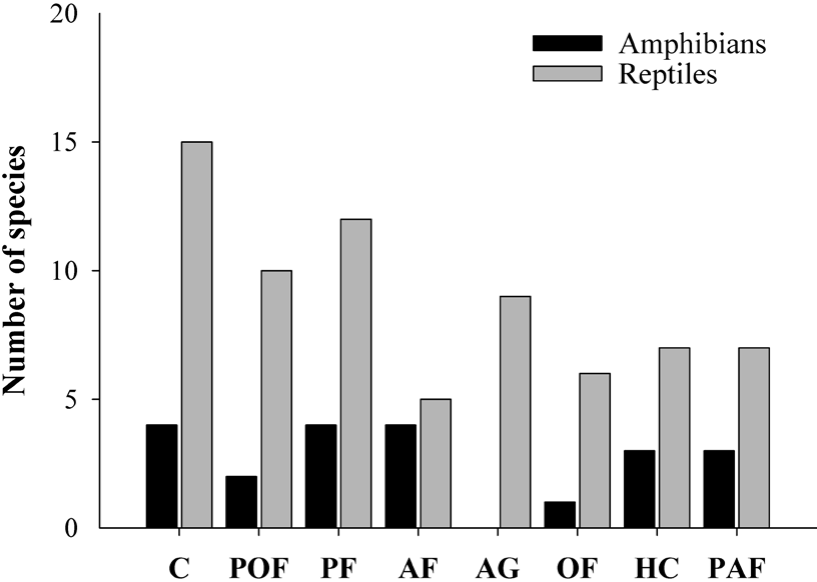
Species richness. Number of amphibian and reptile species by habitat type (Cropland = C, Pine-Oak forest = POF, Pine forest = PF, *Abies* forest = AF, Alpine grassland = AG, Oak forest = OF, Human constructions = HC, Pine-*Alnus* forest = PAF).

We identified eight different habitats (community vegetation and human modification types) occupied by amphibians and reptiles in LMNP: Oak forest (OF), Pine forest (PF), *Abies* forest (AF), Pine-Oak forest (POF), Pine-*Alnus* forest (PAF), Alpine grassland (AG), Human constructions (HC), and Cropland (C). We excluded the AG habitats from amphibian β_sør_ analysis, because, no species were recorded at those elevations. The vegetation communities inhabited by the most amphibians were *Abies* forest, Pine forest, and croplands (four species each). While, the most commonly occupied habitats for reptiles were croplands (15 species), Pine forest (13), and Pine-Oak forest (10) (Fig. 2).

The average dissimilarity for amphibians, was 0.60±0.29 (mean ± 1SD). The highest average of taxonomic replacement was recorded in AF (0.86±0.16), where three plethodontid salamander species were found exclusively in this habitat. In contrast, PF had the lowest dissimilarity (0.42±0.23) among habitat types, with no species unique to the habitat (Table 2). The average dissimilarity for reptiles was lower than that for amphibians (0.40±0.13). The highest dissimilarity value for reptiles was in HC (0.47±0.07), and the lowest in POF (0.30±0.11; Table 3). Croplands had the highest number of unique reptile species (3), followed by AG with a single unique species. The average taxonomic turnover for reptiles and amphibians together (herpetofauna) was 0.46±0.13, and the highest and lowest herpetofaunal turnover rates by habitat type (AF and PF respectively) were the same as found for amphibians alone (Table 4).

**Table 2.** Sørensen pairwise dissimilarity (β_sør_) among vegetation types for the amphibians of LMNP. The average β_sør_ for vegetation types and regional β_sør_ values are shown with one standard deviation. Note that the Alpine grassland was excluded because no amphibian species were recorded in this habitat.

Habitat (unique species)	Pine-Oak forest	Pine forest	*Abies* forest	Oak forest	Human constructions	Pine-*Alnus* forest	Cropland
Pine-Oak forest (0)							
Pine forest (0)	0.33						
*Abies* forest (3)	0.67	0.75					
Oak forest (0)	1.00	0.60	1.00				
Human constructions (0)	1.00	0.43	1.00	0.50			
Pine-*Alnus* forest (0)	0.20	0.14	0.71	0.50	0.67		
Cropland (0)	0.67	0.25	1.00	0.60	0.14	0.43	
Average β_sør_	0.64 (±0.33)	0.42 (±0.23)	0.86 (±0.16)	0.70 (±0.24)	0.62 (±0.34)	0.44 (±0.24)	0.51 (±0.31)
Regional β_sør_	0.60 (±0.29)						

**Table 3.** Sørensen pairwise dissimilarity (β_sør_) among vegetation types for the reptiles of LMNP. The average β_sør_ for vegetation types and regional β_sør_ values are showed with one standard deviation.

**Habitat (unique species)**	**Pine-Oak forest**	**Pine forest**	***Abies* forest**	**Alpine grassland**	**Oak forest**	**Human constructions**	**Pine-*Alnus* forest**	**Cropland**
Pine-Oak forest (0)								
Pine forest (0)	0.09							
*Abies* forest (0)	0.33	0.41						
Alpine grassland (1)	0.26	0.24	0.43					
Oak forest (0)	0.38	0.44	0.27	0.47				
Human constructions (0)	0.41	0.47	0.50	0.50	0.54			
Pine-*Alnus* forest (0)	0.38	0.33	0.45	0.20	0.50	0.54		
Cropland (3)	0.28	0.26	0.60	0.50	0.52	0.36	0.62	
Average β_sør_	0.30 (±0.11)	0.32 (±0.14)	0.43 (±0.11)	0.37 (±0.13)	0.45 (±0.09)	0.47 (±0.07)	0.43 (±0.14)	0.45 (±0.15)
Regional β_sør_	0.40 (±0.13)							

**Table 4.** Sørensen pairwise dissimilarity (β_sør_) among vegetation types for the herpetofauna of LMNP. The average β_sør_ for vegetation types and regional β_sør_ values are showed with one standard deviation.

Habitat (unique species)	Pine-Oak forest	Pine forest	*Abies* forest	Alpine grassland	Oak forest	Human constructions	Pine-*Alnus* forest	Cropland
Pine-Oak forest (0)								
Pine forest (0)	0.14							
*Abies* forest (3)	0.43	0.52						
Alpine grassland (1)	0.33	0.36	0.56					
Oak forest (0)	0.47	0.48	0.50	0.50				
Human constructions (0)	0.55	0.46	0.68	0.58	0.53			
Pine-*Alnus* forest (0)	0.33	0.28	0.56	0.33	0.50	0.58		
Cropland (1)	0.35	0.26	0.71	0.57	0.54	0.31	0.57	
Average β_sim_	0.37 (±0.13)	0.36 (±0.14)	0.56 (±0.10)	0.46 (±0.11)	0.50 (±0.02)	0.53 (±0.12)	0.45 (±0.13)	0.47 (±0.17)
Regional β_sim_	0.46 (±0.13)							

## Discussion

Mexico has 864 species of reptiles and 376 species of amphibians (Flores-Villela and García-Vázquez 2014, Parra-Olea and Flores-Villela 2014). The central mountain region is highly biodiverse and hosts 217 reptiles and131 amphibians; this represents 29% of the Mexican herpetofauna (Flores-Villela et al. 2010). According to our results, 2.3% of the total Mexican herpetofauna and 6.8% of that of the central mountain region is found in LMNP. Most importantly, 89% of the herpetofauna (17 species of reptiles and eight amphibians) in LMNP are endemic to Mexico, and *Pseudoeuryceagadovii* is endemic to this specific volcanic region (Wilson and Johnson 2010). According to Flores-Villela and García-Vázquez (2014), Tlaxcala is the state with the lowest diversity of reptiles in Mexico (31 species), and only 16 amphibian species have been reported in this state (Parra-Olea and Flores-Villela 2014). This means that LMNP is home to more than 56% and 61% of the amphibian and reptile species, respectively, that have been documented in entire state of Tlaxcala. The state of Puebla is different due to the high diversity of herpetofauna there, combined with a larger overall area and wider diversity of ecosystems than Tlaxcala. For that reason, the reptile and amphibian species inhabiting LMNP represent only 9.3% and 12.5%, respectively, of Puebla’s herpetofauna in accordance with the hypotheses of Flores-Villela and García-Vázquez (2014) and Parra-Olea and Flores-Villela (2014). Additionally, it has been proposed by distribution models that *Crotalusintermedius* could inhabit LMNP (Paredes-García et al. 2011), nevertheless this hypothesis has been not corroborated by field work or *in situ* observations, and the nearest records to LMNP are more than 13 km of straight line in a xerophytic scrub habitat (Sánchez-Herrera 1980, Sánchez-Herrera and López-Ortega 1987, Campbell and Lamar 2004).

LMNP plays an important role in Tlaxcala’s herpetofaunal preservation. First, this small area (~8.3% of the total length of the state) hosts more than 60% of the herpetofauna known from the entire state. Second, it is the largest protected area in the state (CONANP 2018). Third, it is a refuge for biodiversity because it is an isolated volcano surrounded primarily by croplands, cattle fields, and human constructions (Villers-Ruiz et al. 2006, Castro-Pérez and Tucker 2009). Therefore, we believe that LMNP has to be considered the most important “conservation island” of Tlaxcala. Despite the protected designation of LMNP, 60% of the protected area has been disturbed and the biodiversity is affected by such activities as deforestation, illegal logging, extraction of moss, cattle, induced fire, and agriculture (Díaz-Ojeda 1992, Vargas-Márquez 1997, Villers-Ruiz and López-Blanco 2004, Rojas-García and Villers-Ruíz 2008). All of these activities endanger the permanence of LMNP’s herpetofauna. Moreover, global reptile diversity is already imperiled due to the rise of environmental temperature (Sinervo et al. 2010). Warmer temperatures restrict the activities (compromise fitness) of reptiles and could cause species extinction and promote distributional shifts. Montane and viviparous species will be most affected by rising temperature. High elevation taxa with lower thermal requirements may become compromised due to the impossibility of expanding their altitudinal distribution interval to less hot areas. Conversely species of lower elevations may expand their altitudinal distribution to cooler areas (Sinervo et al. 2010).

Analysis of herpetofaunal habitat use provides insight to determine high diversity sites in LMNP that may warrant special attention. *Abies* forest has the highest level of taxonomic replacement in addition to hosting the greatest diversity of plethodontid species (four) in LMNP, highlighting the importance of this forest in future conservation plans. Also, the protection of Pine-Oak forest, Pine forest, and Oak forest communities is very important, due to presented high taxonomic turnovers in the two groups of organisms and in the interaction as herpetofaunal analysis. In addition, these habitats are under degradation and pressure from illegal logging, cattle grazing, and fire. According to Koleff and Soberón (2008), amphibians demonstrate a level of endemism and geographical rarity far higher than other vertebrate groups in Mexico, followed by reptiles, with both groups showing the highest β-diversity values of terrestrial vertebrates. Similarly, we found the patterns of taxonomic turnover (β-diversity) of amphibians and reptiles in LMNP to be high and mirroring the general patterns of Mexican fauna (see Espinosa et al. 2008, Koleff and Soberón 2008, Morrone 2014). The high taxonomic turnover of these two groups at LMNP, can be explained by the environmental gradient generated by the altitudinal range, more than the size of the area *per se*. Altitudinal and climatic variation shapes the physiological tolerances of the species (Janzen 1967, Koleff and Soberón 2008), which may restrict some herpetofauna to specific biomes, and these dispersal restrictions can result in small distribution areas. These patterns have important implications for the understanding of the structure of the herpetofaunal community, and should be used to inform and improve conservation strategies. Because of the limited distribution of some species in LMNP (e.g. the three salamanders exclusive to *Abies* forest), small areas that do not include all the habitat types might under-represent the species richness of this protected area.

## Conclusion

This study evaluates the richness and diversity of both protected and disturbed areas in the highly diverse central Mexico region; it provides valuable information on biodiversity to determine priority areas to consider for future management protection. More than 17% of the species (five) registered in LMNP are listed in the IUCN Red List, and 35% have a high EVS vulnerability score (10); despite this, only 60% of these amphibians and reptiles are protected by Mexican law. Paradoxically, 88% of amphibians and 89% of reptiles inhabiting this heavily disturbed and protected area are endemic to Mexico.

In addition, after three studies focusing on the herpetofauna of LMNP since 1978, we found four species previously unreported from the protected area; but, were unable to find another five species previously reported from there. The absence of vouchers, photos, or precise information makes it difficult to determine if these are legitimate records or if it was a case of misidentification of these five species. The worst-case scenario would be that these are incidents of short-term local extinctions (40 years) in a natural protected area. LMNP was decreed a protected area 80 years ago; nevertheless, the issues mentioned previously still have an impact on the biodiversity and the natural environment. In addition, the lack of security in LMNP limits research activities in the most important “conservation island” in Tlaxcala. Urgent actions to promote protection and preservation of the diversity in LMNP are necessary. We feel that these protective actions must involve the lowland communities, offering options to stop the high exploitation of natural resources and to demystify and promote the ecological importance of these two groups of vertebrates. Also, any policies should preserve the geographical connectedness of protected areas (biological corridors) to increase the possibility of exchange of the different vertebrates and vegetation from area to area.

## Supplementary materials

Supplementary material 1 Tables S1–S6 Data type: measurement This dataset is made available under the Open Database License (http://opendatacommons.org/licenses/odbl/1.0/). The Open Database License (ODbL) is a license agreement intended to allow users to freely share, modify, and use this Dataset while maintaining this same freedom for others, provided that the original source and author(s) are credited.Aníbal H. Díaz de la Vega-Pérez, Víctor H. Jiménez-Arcos, Eric Centenero-Alcalá, Fausto R. Méndez-de la Cruz, Andre Ngo
