# Supplementary material for: Diversity and conservation of amphibians and reptiles of a protected and heavily disturbed forest of central Mexico
Source: Zookeys. 2019 Mar 14;830:111–25. doi: 10.3897/zookeys.830.31490 (PMC6428800; doi:10.3897/zookeys.830.31490)
Supplement: Supplementary material 1 [file zookeys-830-111-s001.docx]

Table S1. Taxonomic turnover (β_sim_) among vegetation types for amphibians of LMNP. The average β_sim_ for vegetation types and regional β_sim_ values are shown with one standard deviation. Note the Alpine grassland was excluded because no amphibian species were recorded in this habitat.

| Habitat  (unique species) | Pine-Oak  forest | Pine forest | *Abies* forest | Oak forest | Human constructions | Pine-*Alnus* forest | Cropland |
| --- | --- | --- | --- | --- | --- | --- | --- |
| Pine-Oak forest (0) |  |  |  |  |  |  |  |
| Pine forest (0) | 0.00 |  |  |  |  |  |  |
| *Abies* forest (3) | 0.50 | 0.75 |  |  |  |  |  |
| Oak forest (0) | 1.00 | 0.00 | 1.00 |  |  |  |  |
| Human constructions (0) | 1.00 | 0.33 | 1.00 | 0.00 |  |  |  |
| Pine-*Alnus* forest (0) | 0.00 | 0.00 | 0.67 | 0.00 | 0.67 |  |  |
| Cropland (0) | 0.50 | 0.25 | 1.00 | 0.00 | 0.00 | 0.33 |  |
| Average β_sør_ | 0.50  (±0.45) | 0.22  (±0.23) | 0.82  (±0.21) | 0.33  (±0.52) | 0.50  (±0.46) | 0.28  (±0.33) | 0.35  (±0.37) |
| Regional β_sør_ | 0.43  (±0.40) |  |  |  |  |  |  |

Table S2. Differences in richness by nesting (β_nes_) among vegetation types for amphibians of LMNP. The average β_nes_ for vegetation types and regional β_nes_ values are shown with one standard deviation. Note the Alpine grassland was excluded because no amphibian species were recorded in this habitat.

| Habitat  (unique species) | Pine-Oak  forest | Pine forest | *Abies* forest | Oak forest | Human constructions | Pine-*Alnus* forest | Cropland |
| --- | --- | --- | --- | --- | --- | --- | --- |
| Pine-Oak forest (0) |  |  |  |  |  |  |  |
| Pine forest (0) | 0.333 |  |  |  |  |  |  |
| *Abies* forest (3) | 0.17 | 0.00 |  |  |  |  |  |
| Oak forest (0) | 0.00 | 0.60 | 0.00 |  |  |  |  |
| Human constructions (0) | 0.00 | 0.10 | 0.00 | 0.50 |  |  |  |
| Pine-*Alnus* forest (0) | 0.20 | 0.14 | 0.04 | 0.50 | 0.00 |  |  |
| Cropland (0) | 0.17 | 0.00 | 0.00 | 0.60 | 0.14 | 0.10 |  |
| Average β_sør_ | 0.14  (±0.13) | 0.19  (±0.23) | 0.04  (±0.06) | 0.37  (±0.29) | 0.12  (±0.19) | 0.16  (±0.18) | 0.17  (±0.22) |
| Regional β_sør_ | 0.17  (±0.21) |  |  |  |  |  |  |

Table S3. Taxonomic turnover (β_sim_) among vegetation types for reptiles of LMNP. The average β_sim_ for vegetation types and regional β_sim_ values are shown with one standard deviation.

| Habitat  (unique species) | Pine-Oak forest | Pine forest | *Abies* forest | Alpine grassland | Oak forest | Human constructions | Pine-*Alnus* forest | Cropland |
| --- | --- | --- | --- | --- | --- | --- | --- | --- |
| Pine-Oak forest (0) |  |  |  |  |  |  |  |  |
| Pine forest (0) | 0.00 |  |  |  |  |  |  |  |
| *Abies* forest (0) | 0.00 | 0.00 |  |  |  |  |  |  |
| Alpine grassland (1) | 0.22 | 0.11 | 0.20 |  |  |  |  |  |
| Oak forest (0) | 0.17 | 0.17 | 0.20 | 0.33 |  |  |  |  |
| Human constructions (0) | 0.29 | 0.29 | 0.40 | 0.43 | 0.50 |  |  |  |
| Pine-*Alnus* forest (0) | 0.17 | 0.00 | 0.40 | 0.00 | 0.50 | 0.50 |  |  |
| Cropland (3) | 0.10 | 0.17 | 0.20 | 0.33 | 0.17 | 0.00 | 0.33 |  |
| Average β_sør_ | 0.13  (±0.11) | 0.10  (±0.11) | 0.20  (±0.16) | 0.23  (±0.15) | 0.29  (±0.15) | 0.34  (±0.18) | 0.27  (±0.22) | 0.19  (±0.12) |
| Regional β_sør_ | 0.22  (±0.16) |  |  |  |  |  |  |  |

Table S4. Differences in richness by nesting (β_nes_) among vegetation types for reptiles of LMNP. The average β_nes_ for vegetation types and regional β_nes_ values are shown with one standard deviation.

| Habitat  (unique species) | Pine-Oak forest | Pine forest | *Abies* forest | Alpine grassland | Oak forest | Human constructions | Pine-*Alnus* forest | Cropland |
| --- | --- | --- | --- | --- | --- | --- | --- | --- |
| Pine-Oak forest (0) |  |  |  |  |  |  |  |  |
| Pine forest (0) | 0.09 |  |  |  |  |  |  |  |
| *Abies* forest (0) | 0.33 | 0.41 |  |  |  |  |  |  |
| Alpine grassland (1) | 0.04 | 0.13 | 0.23 |  |  |  |  |  |
| Oak forest (0) | 0.21 | 0.28 | 0.07 | 0.13 |  |  |  |  |
| Human constructions (0) | 0.13 | 0.19 | 0.10 | 0.07 | 0.04 |  |  |  |
| Pine-*Alnus* forest (0) | 0.21 | 0.33 | 0.05 | 0.20 | 0.00 | 0.04 |  |  |
| Cropland (3) | 0.18 | 0.09 | 0.40 | 0.17 | 0.36 | 0.36 | 0.29 |  |
| Average β_sør_ | 0.17  (±0.10) | 0.22  (±0.13) | 0.23  (±0.6) | 0.14  (±0.07) | 0.16  (±0.13) | 0.13  (±0.11) | 0.16  (±0.13) | 0.26  (±0.12) |
| Regional β_sør_ | 0.18  (±0.12) |  |  |  |  |  |  |  |

Table S5. Taxonomic turnover (β_sim_) among vegetation types for herpetofauna of LMNP. The average β_sim_ for vegetation types and regional β_sim_ values are shown with one standard deviation.

| Habitat  (unique species) | Pine-Oak forest | Pine forest | *Abies* forest | Alpine grassland | Oak forest | Human constructions | Pine-*Alnus* forest | Cropland |
| --- | --- | --- | --- | --- | --- | --- | --- | --- |
| Pine-Oak forest (0) |  |  |  |  |  |  |  |  |
| Pine forest (0) | 0.00 |  |  |  |  |  |  |  |
| *Abies* forest (3) | 0.33 | 0.33 |  |  |  |  |  |  |
| Alpine grassland (1) | 0.22 | 0.11 | 0.56 |  |  |  |  |  |
| Oak forest (0) | 0.29 | 0.14 | 0.43 | 0.43 |  |  |  |  |
| Human constructions (0) | 0.50 | 0.30 | 0.67 | 0.56 | 0.43 |  |  |  |
| Pine-*Alnus* forest (0) | 0.22 | 0.00 | 0.56 | 0.33 | 0.43 | 0.56 |  |  |
| Cropland (1) | 0.17 | 0.19 | 0.56 | 0.33 | 0.14 | 0.00 | 0.33 |  |
| Average β_sør_ | 0.25  (±0.15) | 0.15  (±0.13) | 0.49  (±0.13) | 0.36  (±0.17) | 0.33  (±0.14) | 0.43  (±0.22) | 0.35  (±0.20) | 0.25  (±0.18) |
| Regional β_sør_ | 0.33  (±0.19) |  |  |  |  |  |  |  |

Table S6. Differences in richness by nesting (β_nes_) among vegetation types for herpetofauna of LMNP. The average β_nes_ for vegetation types and regional β_nes_ values are shown with one standard deviation.

| Habitat  (unique species) | Pine-Oak forest | Pine forest | *Abies* forest | Alpine grassland | Oak forest | Human constructions | Pine-*Alnus* forest | Cropland |
| --- | --- | --- | --- | --- | --- | --- | --- | --- |
| Pine-Oak forest (0) |  |  |  |  |  |  |  |  |
| Pine forest (0) | 0.14 |  |  |  |  |  |  |  |
| *Abies* forest (3) | 0.10 | 0.19 |  |  |  |  |  |  |
| Alpine grassland (1) | 0.11 | 0.25 | 0.00 |  |  |  |  |  |
| Oak forest (0) | 0.19 | 0.34 | 0.07 | 0.07 |  |  |  |  |
| Human constructions (0) | 0.05 | 0.16 | 0.02 | 0.02 | 0.10 |  |  |  |
| Pine-*Alnus* forest (0) | 0.11 | 0.28 | 0.00 | 0.00 | 0.07 | 0.02 |  |  |
| Cropland (1) | 0.19 | 0.07 | 0.16 | 0.24 | 0.40 | 0.31 | 0.24 |  |
| Average β_sør_ | 0.13  (±0.05) | 0.20  (±0.09) | 0.08  (±0.08) | 0.10  (±0.11) | 0.18  (±0.14) | 0.10  (±0.11) | 0.10  (±0.11) | 0.23  (±0.11) |
| Regional β_sør_ | 0.14  (±0.11) |  |  |  |  |  |  |  |
